# Potassium channels contribute to activity‐dependent regulation of dendritic inhibition

**DOI:** 10.14814/phy2.13747

**Published:** 2018-06-25

**Authors:** Jeremy T. Chang, Michael J. Higley

**Affiliations:** ^1^ Department of Neuroscience Program in Cellular Neuroscience, Neurodegeneration and Repair Kavli Institute Yale School of Medicine New Haven Connecticut

**Keywords:** Calcium, dendrite, GABA

## Abstract

GABAergic inhibition plays a critical role in the regulation of neuronal activity. In the neocortex, inhibitory interneurons that target the dendrites of pyramidal cells influence both electrical and biochemical postsynaptic signaling. Voltage‐gated ion channels strongly shape dendritic excitability and the integration of excitatory inputs, but their contribution to GABAergic signaling is less well understood. By combining 2‐photon calcium imaging and focal GABA uncaging, we show that voltage‐gated potassium channels normally suppress the GABAergic inhibition of calcium signals evoked by back‐propagating action potentials in dendritic spines and shafts of cortical pyramidal neurons. Moreover, the voltage‐dependent inactivation of these channels leads to enhancement of dendritic calcium inhibition following somatic spiking. Computational modeling reveals that the enhancement of calcium inhibition involves an increase in action potential depolarization coupled with the nonlinear relationship between membrane voltage and calcium channel activation. Overall, our findings highlight the interaction between intrinsic and synaptic properties and reveal a novel mechanism for the activity‐dependent regulation of GABAergic inhibition.

## Introduction

Inhibition in the neocortex is primarily mediated by the neurotransmitter gamma‐aminobutyric acid (GABA) through synaptic contacts made by interneurons. These synapses are distributed across the entire somatodendritic arbor and work to counteract excitatory glutamatergic input. GABAergic synapses that target the axon initial segment and soma exert a strong influence on somatic voltage, and consequently play important roles in regulating the generation and timing of action potentials (Pouille and Scanziani 2001; Wehr and Zador 2003; Zhu et al. 2004; Higley and Contreras 2006). However, the vast majority of inhibitory inputs are formed onto pyramidal cell dendrites (Beaulieu and Somogyi 1990), and the role of dendrite‐targeting inhibition has been an area of growing interest (Miles et al. 1996; Lovett‐Barron et al. 2012; Bloss et al. 2016).

One important function of dendritic inhibition, in addition to action potential regulation, is the regulation of dendritic calcium signals which are thought to play an instructive role in synaptic plasticity (Tsubokawa and Ross 1996; Palmer et al. 2012; Chiu et al. 2013). Recent reports in the neocortex and hippocampus have described varying efficacy of dendritic calcium inhibition, ranging from spatial compartmentalization within individual spines to complete abolition of actively back‐propagating action potentials (bAPs)(Kanemoto et al. 2011; Chiu et al. 2013; Marlin and Carter 2014; Stokes et al. 2014; Mullner et al. 2015). The mechanisms underlying the heterogeneity of previous findings remain unclear, but one contributing factor may be variation in intrinsic dendritic properties, like voltage‐dependent channels, whose impact on GABAergic inhibition of bAPs is not well understood. Indeed, earlier work in hippocampal neurons suggested that the inhibition of bAP‐evoked dendritic calcium signaling may be inversely correlated with the magnitude of the calcium transient (Mullner et al. 2015), consistent with an interaction between GABAergic potency and dendritic excitability.

The expression of voltage‐gated ion channels within neuronal dendrites regulates cellular excitability and strongly influences synaptic integration (Miller et al. 1985; Shepherd et al. 1985; Cook and Johnston 1999; Poirazi and Mel 2001; Johnston and Narayanan 2008). Potassium channels, including those sensitive to the blocker 4‐aminopyridine (4‐AP), are expressed throughout the dendritic arbors of cortical and hippocampal pyramidal neurons and have been implicated in the regulation of back‐propagating action potentials (bAPs) and excitatory synaptic integration (Hoffman et al. 1997; Ramakers and Storm 2002; Cai et al. 2004; Gasparini 2011; Carrasquillo et al. 2012; Harnett et al. 2013). Interestingly, A‐type Kv4.2 channels have been shown to preferentially colocalize with GABAergic synapses, suggesting they may also play a role in the control of inhibition (Jinno et al. 2005; Burkhalter et al. 2006).

Here, we examine how voltage‐gated potassium channels alter GABAergic inhibition of bAP‐evoked Ca^2+^ signals (ΔCa^2+^) in dendrites of L5 pyramidal neurons in mouse visual cortex. We show that the blockade of these channels enhances both the amplitude of bAP‐evoked ΔCa^2+^ and unexpectedly also the inhibition of bAP‐evoked ΔCa^2+^. We also show that the voltage‐dependent inactivation of these channels gives rise to a scaling of dendritic GABAergic inhibition, such that inhibitory efficacy is enhanced following strong somatic activity. Thus, our findings demonstrate that intrinsic excitability interacts with GABAergic synaptic input to dynamically regulate dendritic Ca^2+^ signaling.

## Materials and Methods

### Ethical approval

All animal handling and experimental procedures were approved by the Yale Institutional Animal Care and Use Committee and in accordance with federal guidelines.

### Slice preparation

For GABA uncaging experiments, subjects were male wild‐type C57‐BL6 mice, ages P30‐40 (Harlan). For optogenetic experiments, subjects were male and female SOM‐Cre mice, ages P30‐40 (IMSR Cat# JAX:013044, RRID:IMSR_JAX:013044). Under isofluorane anesthesia, mice were decapitated and coronal slices (300 *μ*m thick) containing primary visual cortex were cut in ice cold external solution containing (in mM): 110 choline, 25 NaHCO3, 1.25 NaH2PO4, 2.5 KCl, 7 MgCl2, 0.5 CaCl2, 20 glucose, 11.6 sodium ascorbate, and 3.1 sodium pyruvate, bubbled with 95% O2 and 5% CO2. After an incubation period of 20 min at 34°C, slices were transferred to artificial cerebrospinal fluid (ACSF) containing in (mM): 127 NaCl, 25 NaHCO_3_, 1.25 NaH_2_PO_4_, 2.5 KCl, 1 MgCl_2_, 2 CaCl_2_, and 20 glucose bubbled with 95% O_2_ and 5% CO_2_ and maintained at room temperature (20–22°C) for at least 20 min until use.

### Electrophysiology and imaging

Experiments were conducted at room temperature in a submersion type recording chamber. Whole‐cell patch clamp recordings were obtained from regular‐spiking layer 5A pyramidal neurons (500 *μ*m to 600 *μ*m from the pial surface) identified with video infrared‐differential interference contrast. Most cells exhibited an after‐depolarization following somatic action potentials, and cells that fired bursts of spikes after application of 4‐AP were excluded. For current‐clamp recordings, glass electrodes (2–4 MΩ tip resistance) were filled with internal solution containing (in mM): 135 KMeSO3, 10 HEPES, 4 MGCl2, 4 Na2ATP, 0.5 NaGTP, and 10 sodium creatine phosphate, adjusted to pH 7.3 with KOH. For Ca^2+^ imaging experiments, red fluorescent Alexa Fluor‐568 (40 *μ*mol/L) and green fluorescent Ca^2+^‐sensitive Fluo‐5F (300 *μ*mol/L) were included in the pipette solution to visualize cell morphology and changes of intracellular Ca^2+^ concentration, respectively. Electrophysiological recordings were made using a Multiclamp 700B amplifier (Molecular Devices), filtered at 4 kHz, and digitized at 10 kHz. For all recordings, membrane potential was adjusted to −64 mV using current injection through the pipette.

Two‐photon imaging was performed with a custom‐modified Olympus BX51‐WI microscope, including components manufactured by Mike's Machine Company. Fluorophores were excited using 840 nm light from a pulsed titanium‐sapphire laser. Emissions were separated using appropriate optical filters (Chroma, Semrock) and collected by photomultiplier tubes (Hamamatsu). A mechanical shutter was placed in front of the collectors to prevent damage during blue light stimulation. For Ca^2+^ imaging, signals were collected during a 500 Hz line scan across a mushroom spine and neighboring dendritic shaft on the main apical trunk 100 *μ*m to 150 *μ*m from the cell body. Back‐propagating action potentials (bAPs) were evoked using a brief depolarizing current pulse (0.5 msec, 1.5–2.5 nA) through the recording pipette. Trials including bAP alone, IPSP‐bAP, and IPSP alone were interleaved with a 45 sec intertrial interval. In a subset of experiments, trains of action potentials at 50 Hz and 100 Hz were elicited by current pulse injections through the recording pipette, ending 20 msec prior to a single current pulse. In this case, trials including single bAP alone, train‐bAP alone, IPSP‐bAP, train‐IPSP‐bAP, IPSP alone, train‐IPSP, and train alone were interleaved with a 45 sec intertrial interval. Fluorescent traces were computed for individual cells as the average of 10 trials. For all experiments, only one spine/shaft pair was imaged per cell.

Reference frame scans were taken between each acquisition to correct for small spatial drift over time. Ca^2+^ signals were first quantified as changes in green fluorescence from baseline normalized to the average red fluorescence (ΔG/R). To permit comparison of the imaging data across various microscope configurations, we expressed fluorescence changes as the fraction of the G/R ratio measured in saturating Ca^2+^ (ΔG/Gsat).

### Pharmacology

For all GABA uncaging experiments, ACSF included 3 *μ*M CGP‐55845 hydrochloride (Tocris Cat. No. 1248) to block GABA_B_ receptors, 10 *μ*mol/L (R)‐CPP (Tocris Cat. No. 0247) to block NMDA receptors, and 10 *μ*mol/L NBQX disodium salt (Tocris Cat. No. 1044) to block AMPA receptors. For a subset of experiments, the ACSF included 5 mM 4‐aminopyradine (Tocris Cat. No. 0940) or 100 *μ*mol/L picrotoxin (Tocris Cat. No 1128). Local application of 25 mmol/L 4‐AP was achieved using a glass puffer pipette (<2 *μ*m tip) coupled to a Picospritzer. Drugs were ejected continuously with 10–17 psi, and pipettes were position 30–70 *μ*m from the targeted structure at the surface of the slice. In experiments where one‐photon uncaging was performed with local drug application, 10.8 *μ*mol/L RuBi‐GABA was included in the puffer pipette. In a subset of cells, somatic current injections elicited bursts of action potentials in the presence of 4‐AP and were excluded from subsequent analysis.

Visible light‐evoked GABA uncaging was accomplished using RuBi‐GABA (10.8 *μ*mol/L) bath‐applied in the ACSF (Rial Verde et al. 2008). We overfilled the back aperture of the microscope objective (60x, 1.0 NA) with collimated blue light from a fiber‐coupled 473 nm laser. Spherical aberrations due to fiber coupling resulted in a 15–20 *μ*m diameter disc of light at the focal plane centered on the field of view. A brief (0.5 msec) pulse of light (1–2 mW at the sample) reliably evoked uncaging‐evoked uIPSPs. For Ca^2+^ imaging experiments, a blue light photoartifact was corrected by subtracting fluorescence traces on uncaging‐alone trials from those with Ca^2+^ imaging. For all experiments, GABA uncaging occurred 15 msec prior to bAP initiation.

### ChR2 expression and activation

To stimulate SOM‐INs, SOM‐Cre mice were injected 13–23 days prior to slice preparation into the primary visual cortex with recombinant adeno‐associated virus (AAV) driving conditional expression of a ChR2‐eYFP fusion protein under the Ef1a‐promoter (AAV‐DIO‐Ef1a‐ChR2‐EYFP)(UNC Vector Core). Optogenetic stimulation was accomplished using the same light source and path as one‐photon GABA uncaging (see above). Brief (2–3 mW, 0.5 msec) pulses were used to stimulate SOM‐INs 15 msec prior to bAP initiation.

### NEURON modeling

Multicompartment time‐dependent simulations were run using NEURON v7.4 (NEURON, RRID:SCR_005393, available free at http://neuron.med.yale.edu) and analyzed using custom scripts written in Jupyter Notebooks 4.1.0 using Python 3.5.2. We modified a previously published ball and stick model adding two apical dendrites and dividing the main apical dendrite into 100 segments (length 5 *μ*m each) (Chiu et al. 2013). Sodium channels (4 mS/cm^2^) and Hodgkin–Huxley style potassium channels (0.1 mS/cm^2^) were constant throughout the dendrite. A single dendritic spine (1 *μ*m diameter) was attached to the apical dendrite 122.5 *μ*m from the cell body by a neck (1 *μ*m length, 0.07 *μ*m diameter). A GABAergic synapse (utilizing GABAA receptors) was modeled as an exponential synapse with the NEURON Exp2Syn mechanism (Gmax = 2 nS, *τ*1 = 5 msec, *τ*2 = 74 msec), contacting the dendritic shaft located 122.5 *μ*m from the cell body. Chloride reversal potential was set to −70 mV. We modeled an A‐type potassium conductance using a previously published channel definition that fits observed currents in distal dendrites (Migliore et al. 1999; Acker and Antic 2009). The A‐type channel densities were set at 0 at the soma and proximal dendritic segment and increased linearly with distance. Maximum conductance in the distal segment of the dendrite was varied from 10 mS/cm^2^ to 70 mS/cm^2^. A previously published medium voltage‐gated calcium channel was inserted into the dendritic spine and neighboring dendrite (1e^‐7^ mS/cm^2^) such that currents through these channels would minimally impact membrane potential (Polsky et al. 2004). In order to reproduce our experimental conditions, an iterative search was conducted to find a somatic current injection that maintained the somatic resting potential at 64.00 ± 0.001 mV at the cell body for each condition tested. Back‐propagating action potentials were generated by current injection to the somatic compartment, and inhibitory conductance preceded action potentials by 15 msec. Similar to our experiments, we quantified calcium flux over a 100 msec window in order to calculate percent calcium change due to inhibition. Data were generated for fixed time steps (implicit Euler, dt = 0.005 msec). To speed up simulation time, simulations were run in parallel using the built‐in message passing interface of NEURON.

### Data acquisition and analysis

Imaging and physiology data were acquired using custom software written in MATLAB. Offline analysis was performed using custom routines written in MATLAB (MATLAB, RRID:SCR_001622) and IgorPro (Wavemetrics Software, RRID:SCR_000325). Ca^2+^ responses were calculated as the integral of the fluorescence transient over the first 100 msec after bAP initiation. In order to enable comparisons across cells, Ca2 +  inhibition was expressed as in previous studies (Chiu et al. 2013) as (ΔCa2+_Ctl_‐ΔCa2+_Inh_)/ΔCa2+_Ctl_. In general, data were non‐normally distributed. Therefore, all statistical comparisons were made using the nonparametric Wilcoxon‐matched pairs signed rank test in GraphPad Prism version 7.01 (GraphPad Prism, RRID:SCR_002798) unless otherwise noted.

## Results

**Figure 1:**
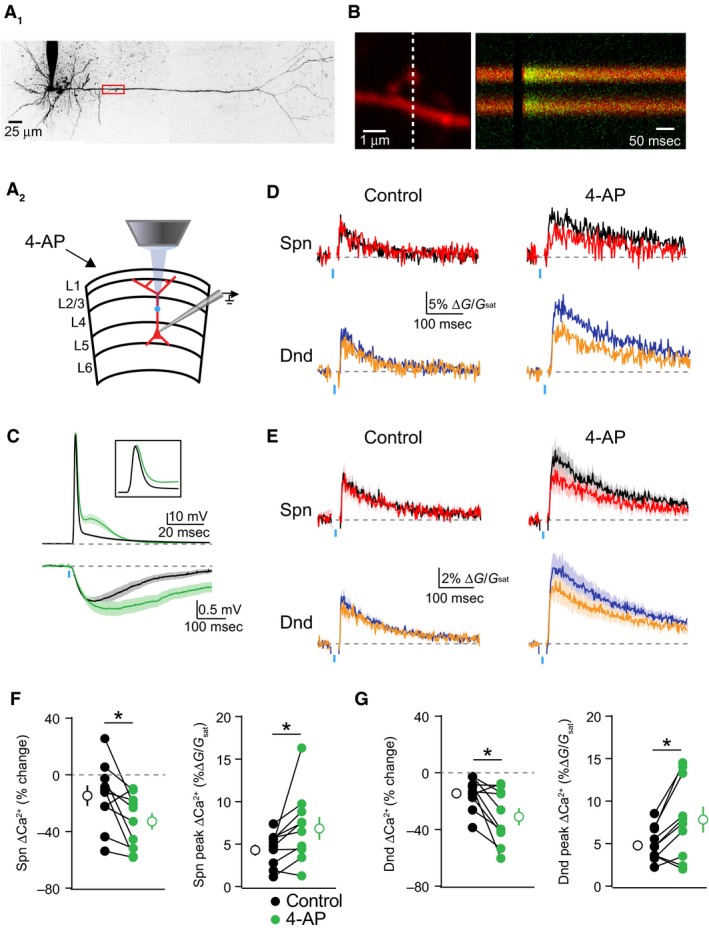
GABAergic inhibition of ΔCa^2+^ is enhanced by blockade of potassium channels. (A) Whole‐cell patch recordings were performed in L5PNs of visual cortex. Ca^2+^ imaging and GABA uncaging were performed along the proximal apical dendrite, as shown in the example cell (A1) and schematic (A2). (B) Example spine‐dendrite pair and the associated line‐scanned response to a bAP. (C) Average ± SEM somatic voltage recorded before (black) and after (green) treatment with 4‐AP for action potentials (upper traces) and uncaging‐evoked uIPSPs (lower traces). (D) Example bAP‐evoked ΔCa^2+^ for the apical dendritic region shown in (B) for bAP alone (black, blue) or paired with GABA uncaging (red, orange) for the spine and neighboring dendrite, before (left) and after (right) treatment with 5 mM 4‐AP. (E) Average (*n* = 10) ΔCa^2+^ for the population of imaged spines and dendrites, colors as in (D). (F–G) Population data (*n* = 10) showing the magnitude of ΔCa^2+^ inhibition and peak bAP‐evoked ΔCa^2+^ for spines (F) and neighboring dendrites (G) before (black) and after (green) treatment with 5 mM 4‐AP (Wilcoxon matched‐pairs signed rank test, *P < 0.05).

**Figure 2:**
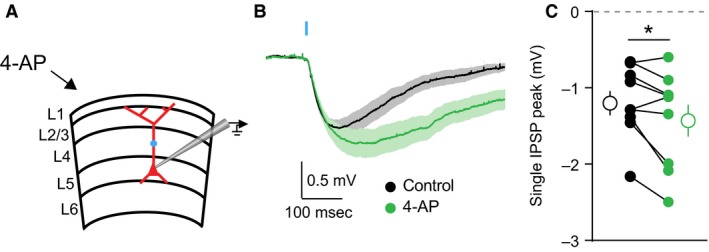
Blocking potassium channels increases GABAergic uIPSPs. (A) Schematic of recording configuration, illustrating location of GABA uncaging. (B) Average ± SEM (*n* = 9) IPSPs in control saline (black) or after bath application of 4‐AP (green). (C) Population data (*n* = 9) showing the magnitude of uIPSP enhancement after 4‐AP (Wilcoxon matched‐pairs signed rank test, *P* > 0.05).

In order to investigate the impact of potassium channels on dendritic inhibition, we performed simultaneous whole‐cell current clamp recordings and two‐photon calcium imaging of bAP‐evoked dendritic Ca^2+^ transients in layer 5 pyramidal neurons (L5PNs) of mouse visual cortex (Fig. 1A). Ca^2+^ signals were measured in dendritic spines and neighboring shafts along the primary apical dendrite, 100–150 *μ*m from the soma (Fig. 1B). We focused on the proximal apical dendrite, as single action potentials reliably propagate through this region. To probe the effects of GABAergic inhibition on ΔCa^2+^, we compared uninhibited ΔCa^2+^ from bAPs induced by somatic current injection with ΔCa^2+^ from bAPs preceded (15 msec) by local (at the imaging site) uncaging of RuBi‐GABA (Rial Verde et al. 2008). To compare observations across different recordings, GABAergic inhibition of ΔCa^2+^ was quantified as in previous studies as (ΔCa2+_Ctl_‐ΔCa2+_Inh_)/ΔCa2+_Ctl_ (Chiu et al. 2013). The magnitude of this Ca^2+^ inhibition was measured before and after bath‐application of the potassium channel blocker 4‐aminopyridine (4‐AP)(Fig. 1A), whereas the resting membrane potential was kept constant (~‐64 mV) using current injection through the pipette. Before 4‐AP, the population of dendritic spines showed a trend toward significant Ca^2+^ inhibition (*P* = 0.07)(*n* = 10 spines), whereas dendritic shafts were significantly inhibited (*P* = 0.005)(*n* = 10 shafts)(Wilcoxon signed rank test). Treatment with 4‐AP broadened the somatic action potential (*P* = 0.0020) but did not increase the action potential amplitude (*P* = 0.49, *n* = 10 cells)(Fig. 1C). 4‐AP application increased the average peak ΔCa^2+^ evoked by a single bAP for both individual spines (*P* = 0.0059)(*n* = 10 spines) and neighboring shafts (*P* = 0.0137)(*n* = 10 shafts)(Fig. 1D–G). Moreover, 4‐AP significantly increased the amplitude of the uncaging‐evoked inhibitory postsynaptic potential (uIPSP, Fig. 1C, Fig. 2, *P* = 0.0391, *n* = 9 cells) and enhanced the average GABAergic inhibition of ΔCa^2+^ for both individual spines (*P* = 0.0195)(*n* = 10 spines) and neighboring shafts (*P* = 0.0098)(*n* = 10 shafts)(Fig. 1D–G). Indeed, after 4‐AP, the populations of both dendritic spines (*P* = 0.005) and dendritic shafts (*P* = 0.005) were significantly inhibited (Wilcoxon signed rank test). These results were not observed when slices were pretreated with the GABA_A_R antagonist picrotoxin (data not shown).

**Figure 3:**
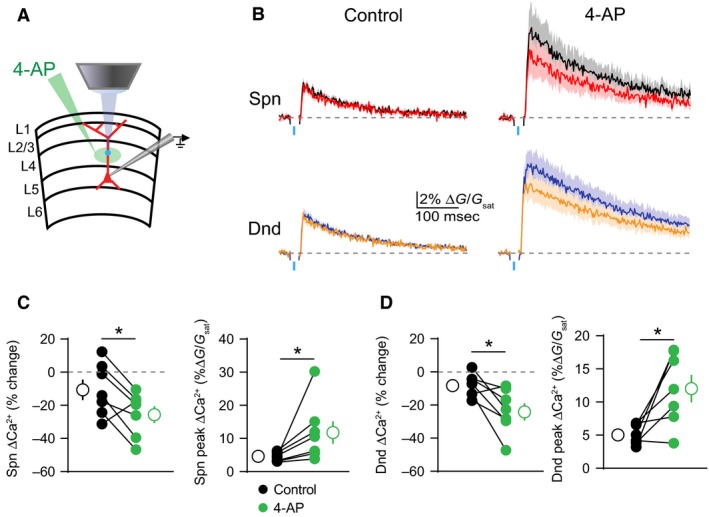
GABAergic inhibition of ΔCa^2+^ is enhanced by local blockade of potassium channels. (A) Schematic of recording and imaging configuration, illustrating location of puffed 4‐AP. (B) Average ± SEM (*n* = 7) Ca^2+^ transients for bAP alone or paired with GABA uncaging before (left) and after (right) application of 4‐AP to the proximal apical dendrite (colors as in Fig. 1). (C–D) Population data (*n* = 7) showing the magnitude of Ca^2+^ inhibition and peak bAP‐evoked Ca^2+^ for spines (C) and neighboring dendritic shafts (D) in control (black) and 4‐AP (green) conditions (Wilcoxon matched‐pairs signed rank test, *P < 0.05).

**Figure 4:**
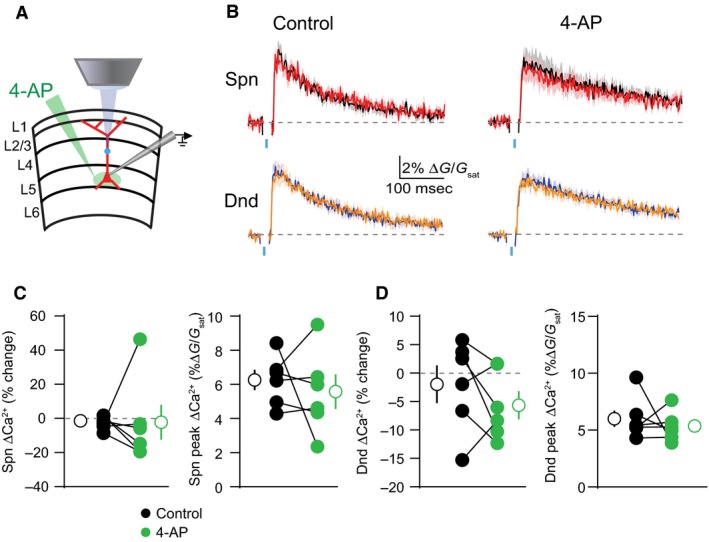
GABAergic inhibition of ΔCa^2+^ in the proximal apical dendrite is not affected by somatic potassium channel blockade. (A) Schematic of recording and imaging configuration, illustrating somatic location of puffed 4‐AP. (B) Average ± SEM (*n* = 6) Ca^2+^ transients for bAP alone or paired with GABA uncaging before (left) and after (right) application of 4‐AP to the soma (colors as in Fig. 1). (C–D) Population data (*n* = 6) showing the magnitude of Ca^2+^ inhibition and peak bAP‐evoked Ca^2+^ for spines (C) and neighboring dendritic shafts (D) in control (black) and 4‐AP (green) conditions (Wilcoxon matched‐pairs signed rank test, *P* > 0.05).

Next, we investigated whether the actions of 4‐AP on dendritic Ca^2+^ inhibition required block of potassium channels near the site of GABAergic input. We used a puffer pipette to locally apply 4‐AP at different locations along the somatodendritic axis. When applied to the proximal apical dendrite (at the site of GABA uncaging), 4‐AP replicated the effects of bath‐application on the magnitude of bAP‐evoked ΔCa^2+^ and its inhibition by GABA. Specifically, 4‐AP increased ΔCa^2+^ in spines (*P* = 0.0156) and neighboring shafts (*P* = 0.0313)(*n* = 7 spines)(Fig. 3A–D). GABAergic inhibition of ΔCa^2+^ was also enhanced in spines (*P* = 0.0469) and neighboring shafts (*P* = 0.0313)(*n* = 7 shafts)(Fig. 3A–D). In contrast, application of 4‐AP to the cell body had no impact on peak ΔCa^2+^ or its inhibition by GABA within the proximal apical dendrite (Fig. 4A–D). Thus, our data suggest that the impact of 4‐AP on GABAergic inhibition of Ca^2+^ is mediated by dendritic potassium channels near the site of synaptic input.

**Figure 5:**
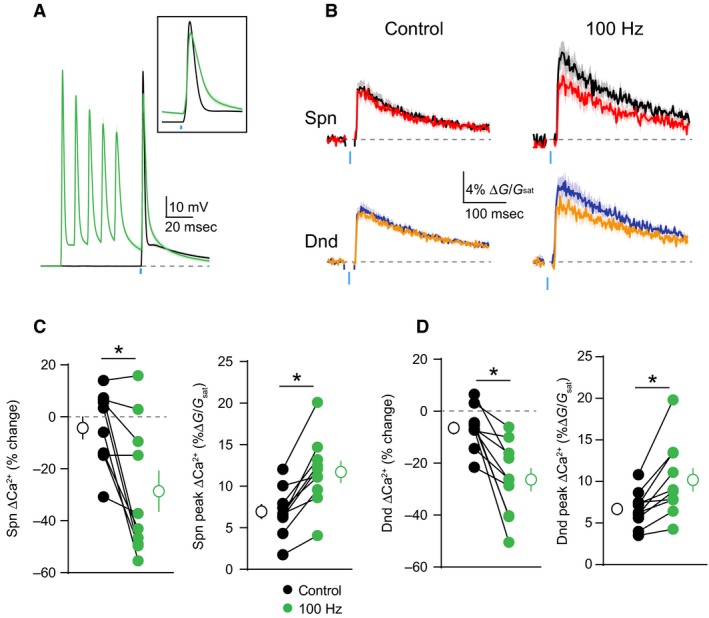
Somatic activity enhances GABAergic inhibition of dendritic ΔCa2 + . (A) Average ± SEM (*n* = 10) of somatic recordings for a single action potential (black) or when preceded 20 msec by a 100 Hz train of action potentials (green). Inset shows change in spike waveform. (B) Average ± SEM (*n* = 10) Ca^2+^ transients for bAP alone or paired with GABA uncaging, presented either singly (left) or following a 100 Hz train of action potentials (right)(colors as in Fig. 1). (C–D) Population data (*n* = 10) showing the magnitude of Ca^2+^ inhibition and peak bAP‐evoked Ca^2+^ for spines (C) and neighboring dendritic shafts (D), presented either singly (black) or following a 100 Hz train of action potentials (green)(Wilcoxon matched‐pairs signed rank test, *P < 0.05).

**Figure 6:**
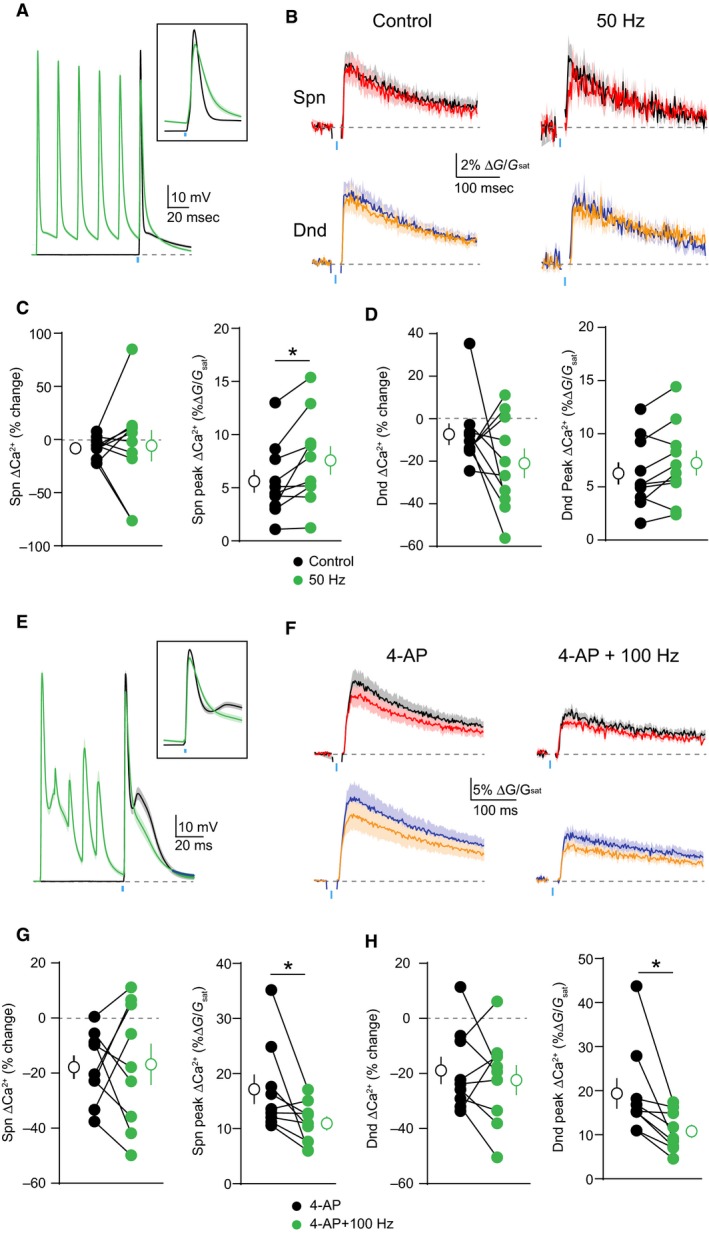
Activity‐dependent enhancement of GABAergic inhibition is frequency‐dependent and occluded by blockade of KA channels. (A) Average ± SEM (*n* = 10) of somatic recordings for a single action potential (black) or when preceded 20 msec by a 50 Hz train of action potentials (green). Inset shows change in spike waveform. (B) Average ± SEM (n = 10) Ca^2+^ transients for bAP alone or paired with GABA uncaging, presented either singly (left) or following a 50 Hz train of action potentials (right)(colors as in Fig. 1). (C–D) Population data (*n* = 10) showing the magnitude of Ca^2+^ inhibition and peak bAP‐evoked Ca^2+^ for spines (C) and neighboring dendritic shafts (D), presented either single (black) or following a 50 Hz train of action potentials (green)(Wilcoxon matched‐pairs signed rank test, *P < 0.05). (E) Average ± SEM (n = 10) of somatic recordings for a single action potential (black) or when preceded 20 msec by a 100 Hz train of action potentials (green), in the presence of 5 mM 4‐AP. (F–H) As in (B‐D) for a 100 Hz preceding train and in the presence of 5 mM 4‐AP (*n* = 10, Wilcoxon matched‐pairs signed rank test, *P < 0.05).

One feature of many potassium channels is their voltage‐dependent inactivation, which limits their conductance during periods of high neuronal activity (Bekkers 2000; Kim et al. 2005). We therefore asked whether this property might enable dendritic GABAergic inhibition to dynamically scale with somatic firing. To test this hypothesis, we compared GABAergic inhibition of ΔCa^2+^ evoked by a bAP alone or preceded 20 msec by a train of 5 bAPs at 100 Hz (Fig. 5A). Similar to 4‐AP, the preceding train significantly enhanced the peak ΔCa^2+^ for both spines (*P* = 0.002)(*n* = 10 spines) and neighboring shafts (*P* = 0.002)(n = 10 shafts) and also enhanced GABAergic inhibition of ΔCa^2+^ for spines (*P* = 0.0059)(*n* = 10 spines) and neighboring shafts (*P* = 0.002)(*n* = 10 shafts)(Fig. 5B–D). In contrast, preceding spike trains at 50 Hz had minimal effect on either ΔCa^2+^ amplitude or GABAergic inhibition (Fig. 6D), indicating that inhibition scales with activity. Importantly, the ability of the 100 Hz train to enhance dendritic inhibition was occluded by prior bath application of 4‐AP (Fig. 6E–H), suggesting that both manipulations target a similar population of dendritic potassium channels.

**Figure 7:**
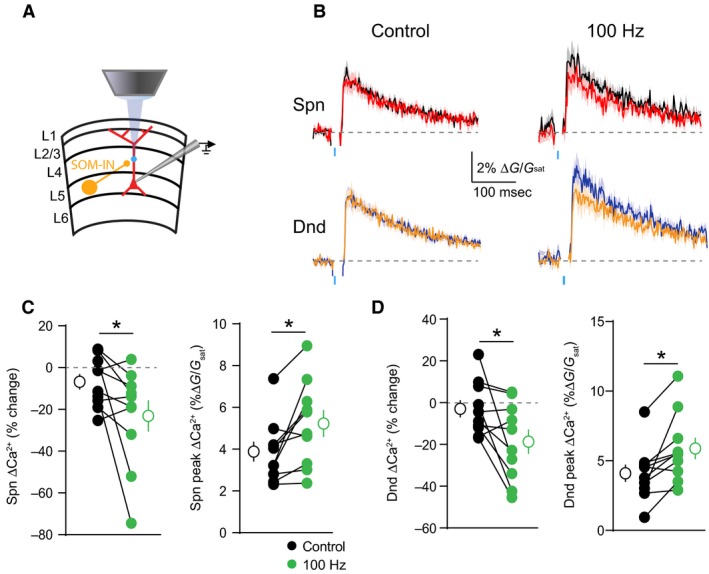
Somatic activity enhances synaptic GABAergic inhibition of ΔCa^2+^. (A) Schematic showing recording and imaging configuration. ChR2 was virally expressed in somatostatin‐containing interneurons (SOM‐INs) and activated with blue light pulses. (B) Average ± SEM (*n* = 10) Ca^2+^ transients for bAP alone or paired with optical stimulation of SOM‐INs, presented either singly (left) or following a 100 Hz train of action potentials (right)(colors as in Fig. 1). (C–D) Population data (*n* = 10) showing the magnitude of Ca^2+^ inhibition and peak bAP‐evoked Ca^2+^ for spines (C) and neighboring dendritic shafts (D), presented either singly (black) or following a 100 Hz train of action potentials (green)(Wilcoxon matched‐pairs signed rank test, *P < 0.05).

We next asked whether activity‐dependent scaling of inhibition could be seen with synaptic GABA release. To test this, we expressed channelrhodopsin‐2 (ChR2) in a subset of dendrite‐targeting cortical interneurons expressing somatostatin (SOM‐INs)(Fig. 7A). Brief pulses of blue light were used to activate SOM‐INs and produce postsynaptic IPSPs. We repeated experiments comparing the GABAergic inhibition of ΔCa^2+^ evoked by a bAP alone or preceded by a 100 Hz train. Before the train, the populations of dendritic spines (*P* = 0.11) and shafts (*P* = 0.33) were not significantly inhibited (Wilcoxon signed rank test). However, as with GABA uncaging, trains of somatic action potentials significantly enhanced the magnitude of ΔCa^2+^ in spines (*P* = 0.0098)(*n* = 10 spines) and neighboring shafts *P* = 0.0059)(*n* = 10 shafts) and led to stronger GABAergic inhibition of ΔCa^2+^ for both spines (*P* = 0.0273)(*n* = 10 spines) and neighboring shafts (*P* = 0.0273)(*n* = 10 shafts)(Fig. 7B–D). Indeed, after the train, the populations of dendritic spines (*P* = 0.009) and shafts (*P* = 0.02) were now significantly inhibited (Wilcoxon signed rank test). Taken together, these results demonstrate that voltage‐dependent potassium channels in the apical dendrite play a key role in shaping the impact of synaptic GABAergic inhibition on bAP‐evoked ΔCa^2+^.

**Figure 8:**
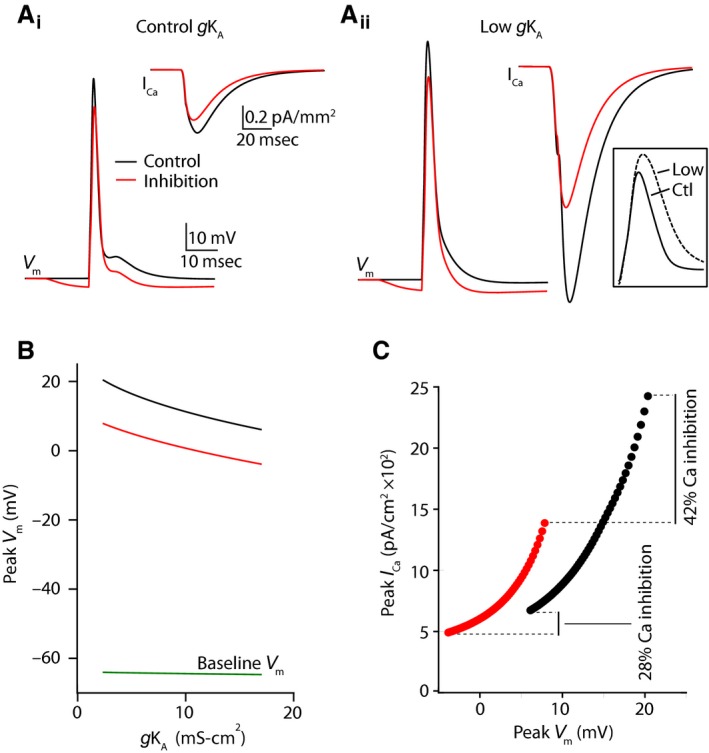
Computational simulations reveal mechanisms underlying potassium channel‐dependent regulation of inhibition. (A) Simulated action potential waveforms and Ca^2+^ currents under control conditions (black) and when preceded 15 msec by a GABAergic IPSP for a control value of gK_A_ (A1) and lowered gK_A_ (A2). Inset shows action potential waveforms for control and lowered gK_A_ values. (B) Relationship between peak membrane potential during the AP and the magnitude of gK_A_ for control conditions (black) and following GABAergic inhibition (red). The baseline resting membrane potential is shown in green. (C) Relationship between peak Ca^2+^ current and peak membrane potential during the AP for control conditions (black) and following GABAergic inhibition (red). Lines traverse varying magnitude of gK_A_ values.

Finally, to examine the biophysical mechanisms underlying the interaction of dendritic potassium channels with GABAergic signaling, we simulated an active dendritic compartment (see Methods) and tested the impact of varying an A‐type potassium conductance (gK_A_) on the magnitude of Ca^2+^ inhibition (Fig. 8). We then varied the maximal dendritic gK_A_ between control (70 mS/cm2 in the distal compartment, 17.0 mS/cm^2^ at the synapse) and low (10 mS/cm2 in the distal compartment, 2.4 mS/cm^2^ at the synapse) conditions, simulating application of 4‐AP, increasing the peak AP amplitude, broadening the AP duration, and enhancing Ca^2+^ inhibition (Fig. 8A). Interestingly, the reduction in peak AP amplitude caused by GABAergic inhibition was not strongly affected by reducing gK_A_ (Fig. 6B). However, we found that the relationship between peak AP amplitude and peak Ca^2+^ current was highly supralinear (Fig. 8C). Thus, a similar amount of GABAergic shift in the peak membrane potential produced a substantially larger inhibition of Ca^2+^ influx when gK_A_ was reduced (Fig. 8C). Our model therefore demonstrates a straightforward biophysical mechanism linking potassium conductance, GABAergic signaling, and Ca^2+^ inhibition in PN dendrites.

## Discussion

Voltage‐gated potassium channels are widely recognized as key modulators of neuronal excitability as well as synaptic integration and plasticity (Hoffman et al. 1997; Magee and Carruth 1999; Losonczy et al. 2008; Carrasquillo et al. 2012; Foeger et al. 2012; Harnett et al. 2013). In the present work, we have described a role for these channels in the regulation of GABAergic control over dendritic Ca^2+^ signaling. Using a combination of electrophysiology, 2‐photon Ca^2+^ imaging, and focal GABA uncaging, we show that blocking potassium channels either pharmacologically or via activity‐dependent inactivation enhances both bAP‐evoked Ca^2+^ influx and GABAergic inhibition of these transients in the apical dendrites of L5PNs. Our results demonstrate that dendritic inhibition is highly regulated by the expression of voltage‐dependent channels near the site of synaptic input.

Importantly, our work addresses the interaction of potassium conductance and GABAergic inhibition in the proximal apical dendrites of layer 5A regular‐spiking neurons. Future studies are necessary to determine if these findings generalize to burst‐firing cells more typically found in layer 5B or to the more distal regions of the apical dendrites. Furthermore, the present experiments were conducted at room temperature to preserved cell health during the long‐duration protocols. However, our previous studies (Chiu et al. 2013) showed that dendritic inhibition is similar at physiological temperatures, suggesting that our results are likely to hold in this regime as well.

Dendritic potassium channels comprise a diverse molecular group, including both Kv1‐, Kv3, and Kv4‐type channels (Serodio et al. 1994; Carrasquillo et al. 2012). The observation that brief trains of APs appear to rapidly inactivate the channels regulating dendritic inhibition suggests a contribution from A‐type conductances, known to be highly sensitive to both membrane depolarization and 4‐AP (Serodio and Rudy 1998; Bekkers 2000; Korngreen and Sakmann 2000; Clark et al. 2008). Several previous studies have implicated A‐type channels in the regulation of both dendritic excitability and glutamatergic synaptic integration. In both CA1 and cortical pyramidal neurons, the presence of A‐type channels limits the spread of voltage between distinct compartments, such as the distal and proximal apical dendrite, regulating both the back propagation of action potentials and the spread of synaptically evoked dendritic spikes (Frick et al. 2003; Cai et al. 2004; Kim et al. 2005, 2007; Losonczy et al. 2008; Harnett et al. 2013). Our study suggests that these channels similarly restrict the efficacy of GABAergic inhibition, enabling A‐type channels to serve as dendritic “shock absorbers”, limiting the impact of synaptic inputs from all sources (Yuste 1997). An intriguing possibility is that voltage‐gated potassium channels asymmetrically regulate excitation and inhibition, potentially leading to moment‐to‐moment alterations of the balance between these opposing drives, a hypothesis whose examination will require additional studies.

In addition to regulating dendritic excitability, voltage‐dependent potassium channels have been implicated in shaping long‐term plasticity of glutamatergic synapses. In particular, studies have focused on spike‐timing dependent plasticity (STDP), where bAPs can potentiate or depress synaptic inputs depending on their relative timing to synaptic activity (Magee and Johnston 1997; Markram et al. 1997). For example, EPSPs in CA1 pyramidal neuron dendrites can inactivate A‐type channels, enhancing the dendritic invasion of somatic action potentials and subsequent plasticity (Hoffman et al. 1997). Recent experimental and computational studies have also suggested a key role for GABAergic inhibition in spike‐timing dependent plasticity (Hayama et al. 2013; Paille et al. 2013; Cichon and Gan 2015; Wilmes et al. 2016). For example, focal activation of GABAergic synapses in CA1 dendrites was shown to convert long‐term potentiation to depression due to negative regulation of dendritic Ca^2+^ influx (Hayama et al. 2013). Together, these various findings suggest that the interaction of NMDARs, A‐type channels, and GABAergic inhibition may strongly contribute to the development and maintenance of cortical circuits.

It is intriguing to speculate that expression patterns of potassium channels may also explain some of the recent diversity in studies examining GABAergic control of dendritic ΔCa^2+^. Previous work showed that inhibition could be highly compartmentalized in layer 2/3 pyramidal neurons, with neighboring spines exhibiting markedly different amounts of inhibition (Chiu et al. 2013). In contrast, other studies have shown that more broad dendritic inhibition can occur in L5PNs and hippocampal CA1 pyramidal neurons (Marlin and Carter 2014; Stokes et al. 2014; Mullner et al. 2015). Differential expression and recruitment of voltage‐gated conductances, such as A‐type channels, would be expected to contribute to the heterogeneity of inhibitory function across cell types.

Our computational modeling provides additional insight into the biophysical mechanism underlying the interaction of potassium channels and GABAergic inhibition of Ca^2+^ influx. We found that decreasing gK_A_ increased the peak depolarization of the AP, producing a supralinear increase in Ca^2+^ current and a subsequent enhancement of Ca^2+^ inhibition. The relationship between action potential waveform and calcium influx has been demonstrated previously in presynaptic terminals (Augustine 1990; Zucker et al. 1991). Our results highlight a similar phenomenon in dendrites and indicate that a major contributor to the potency of GABAergic influence over dendritic Ca^2+^ signaling is the relationship between bAP waveform and Ca^2+^ channel activation. Our results differ from previously published work in hippocampal PNs that found increased Ca^2+^ inhibition for smaller Ca^2+^ transients (Mullner et al. 2015). In contrast, we find that in both experimental and simulation data, GABAergic inhibition is more potent when the Ca^2+^ transient is larger following reduction of potassium channel conductance. The disparate findings likely reflect a complicated relationship between total membrane conductance, transient depolarization, and Ca^2+^ channel activation that may vary between cell types and experimental conditions.

Finally, we found that the likely voltage‐dependent inactivation of potassium channels allows for the enhancement of GABAergic inhibition in the presence of high frequency somatic spike generation. This suggests that dendritic inhibition may exert greater control over Ca^2+^ signaling during periods of high network activity or somatic depolarization, essentially acting as a source of homeostatic control. These findings are consistent with previous experimental and computational studies demonstrating the activity‐dependent amplification of trains of bAPs in L5PN apical dendrites (Larkum et al. 1999; Kim et al. 2005; Grewe et al. 2010). Our results suggest that the dynamic properties of active dendritic conductances enable the alteration of GABAergic inhibition over short millisecond time frames, providing the basis for a context‐dependent, flexible role of GABAergic signaling in shaping biochemical signaling in dendrites.

## Conflict of Interest

The authors declare that they have no competing financial interests.
